# The Mechanisms of Type 2 Diabetes-Related White Matter Intensities: A Review

**DOI:** 10.3389/fpubh.2020.498056

**Published:** 2020-11-17

**Authors:** Jing Sun, Baofeng Xu, Xuejiao Zhang, Zhidong He, Ziwei Liu, Rui Liu, Guangxian Nan

**Affiliations:** ^1^Department of Neurology, China-Japan Union Hospital of Jilin University, Changchun, China; ^2^Department of Neurosurgery, First Hospital of Jilin University, Changchun, China; ^3^Department of Endocrinology, China-Japan Union Hospital of Jilin University, Changchun, China; ^4^Department of Neurosurgery, China-Japan Union Hospital of Jilin University, Changchun, China; ^5^Department of VIP Unit, China-Japan Union Hospital of Jilin University, Changchun, China

**Keywords:** type 2 diabetes mellitus, white matter hyperintensities, mechanism, microvascular complication, review

## Abstract

The continually increasing number of patients with type 2 diabetes is a worldwide health problem, and the incidence of microvascular complications is closely related to type 2 diabetes. Structural brain abnormalities are considered an important pathway through which type 2 diabetes causes brain diseases. In fact, there is considerable evidence that type 2 diabetes is associated with an increased risk of structural brain abnormalities such as lacunar infarcts (LIs), white matter hyperintensities (WMHs), and brain atrophy. WMHs are a common cerebral small-vessel disease in elderly adults, and it is characterized histologically by demyelination, loss of oligodendrocytes, and vacuolization as a result of small-vessel ischemia in the white matter. An increasing number of studies have found that diabetes is closely related to WMHs. However, the exact mechanism by which type 2 diabetes causes WMHs is not fully understood. This article reviews the mechanisms of type 2 diabetes-related WMHs to better understand the disease and provide help for better clinical treatment.

## Introduction

With the increasing aging population in China, the incidence rate of type 2 diabetes among the elderly is also increasing yearly. Accordingly, the metabolic disorders and vascular diseases caused by type 2 diabetes are receiving increasing attention. Considerable evidence shows that type 2 diabetes is closely related to cerebral small vessel diseases, which are a key factor for induction of white matter intensities (WMHs).

WMHs are a silent brain injury that appears around the cerebral ventricles and/or deep subcortical white matter that are seen as high-intensity lesions in T2-weighted and fluid-attenuated inversion recovery (FLAIR) images and as isointense or hypointense on T1-weighted images on magnetic resonance imaging (1). Currently, age and hypertension are considered to be consistent risk factors for WMHs. Studies have shown that diabetic subjects are more prone to having more and larger WMHs than non-diabetic subjects (2–5). A recent review by Del Bene et al. more firmly emphasized the relationship between type 2 diabetes and both the presence and severity of WMHs, although the underlying mechanism is still not fully understood (6).

In the present systematic review, we discuss the possible mechanisms for WMH-related type 2 diabetes to provide new ideas for the prevention and treatment of the condition.

### The Possible Mechanism for Type 2 Diabetes-Related WMHs

The prevalence of WMHs increases from about 5% in people aged 50 years to nearly 100% for people aged 90 years (7). Although WMHs are gradually attracting more attention, their pathogenesis is still poorly understood. Numerous studies have suggested that WMHs originate from the chronic hypoperfusion and multiple factors involved in the pathogenesis of WMHs, such as cerebral blood flow autoregulation, venous collagenosis, impaired cerebrovascular reactivity, and blood–brain barrier disruption.

**Figure 1:**
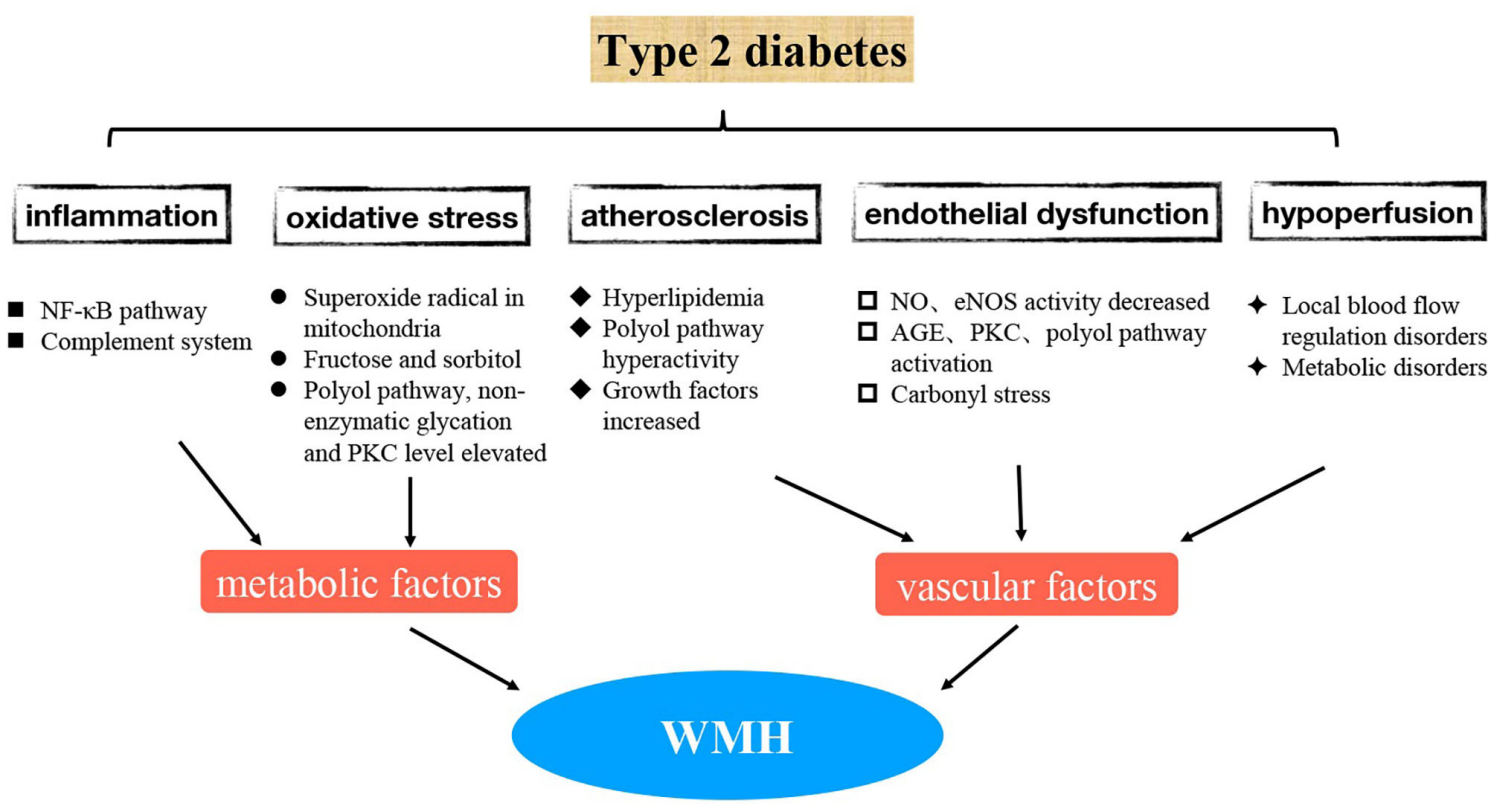
The possible mechanism for WMH-related type 2 diabetes.

At present, many new advances have been made to better understand the mechanism of WMH-related type 2 diabetes. These involve inflammatory response, oxidative stress, endothelial dysfunction, and other aspects (8, 9) (Figure 1). In the future, pathogenesis-targeted treatment may bring new hope to prevent and delay the occurrence and development of diabetic WMHs; hence, it is very important to understand these mechanisms in detail.

1. WMHs are believed to be associated with decreased local cerebral blood perfusion, impaired capillary permeability, and impaired blood-brain barrier (BBB) (10). The precise mechanisms whereby WMHs progress in patients with type 2 diabetes are unclear. However, as WMHs reflect vascular damage, small vessel abnormalities associated with DM could contribute to the formation of WMHs (Figure 1). The associations of DM and continuous measures of hyperglycemia with WMHs can be explained by several mutually non-exclusive mechanisms.

2. Regarding the possible mechanism of WMH pathogenesis, pathology studies have shown that vascular integrity declines first, followed by an increase in BBB permeability, which is mainly caused by endothelial dysfunction (11). Generally, in diabetes, endothelial nitric oxide synthase (eNOS) activity and nitric oxide (NO) production are reduced, resulting in endothelial cell dysfunction and impaired vasodilatation (8). Specifically, hyperglycemia causes cell damage by promoting advanced glycation end products (AGE), activating protein kinase C (PKC), and activating the polyol pathway. Activation of the polyol pathway consumes nicotinamide adenine dinucleotide phosphate (NADPH), which reduces eNOS activity and NO production, causing endothelial cell dysfunction (8). Patients with type 2 diabetes experience some pathologic conditions, such as long-term high blood glucose and multi-substance metabolic disturbance, which damage the blood vessel endothelium in the long term. There are reported associations between soluble intercellular adhesion molecule-1 (sICAM-1), a marker of vascular endothelial damage, and progression of WMHs in patients with type 2 diabetes (12). When damaged, endothelial cells release pro-coagulant molecules such as VWF, PAI-1, and thromboxane A2 and express on their surfaces tissue factor (TF) and adhesion molecules such as P-selectin, E-selectin, vascular adhesion molecular-1 (VCAM-1), and intercellular adhesion molecule-1 (ICAM-1), which mediate the interaction between neutrophils and platelets with the endothelium. Therefore, endothelial dysfunction can promote both pro-inflammatory and pro-coagulant states. A recent study provided novel evidence that hyperglycemia is associated with carbonyl stress in the disruption of the brain endothelial cell barrier dysfunction, a process that is correlated with elevated occludin methylglyoxal glycation, decreased glyoxalase II activity, and reduced GSH-dependent cellular methylglyoxal elimination (13). In conclusion, a variety of pathways and factors contribute to the dysfunction of brain microvascular endothelial cells in type 2 diabetic patients, thereby promoting the occurrence of WMHs. Therefore, the protection of vascular endothelial function may prevent the appearance of WMHs in diabetic patients.

3. Atherosclerosis due to diabetes mellitus is the pathological basis of diabetes mellitus combined with cerebrovascular disease. Insulin deficiency in the body of diabetic patients causes glucose to convert into a large amount of fat, which is broken down into triglycerides and free fatty acids. As a result, cholesterol increases, leading to hyperlipidemia and accelerating arteriosclerosis in diabetic patients. In addition, a previous study has shown that hyperglycemia-induced polyol pathway hyperactivity may play an important part in the development of diabetic atherosclerosis (8). Additionally, diabetic patients contain more hyaline substance in their blood vessels than non-diabetic patients, and their blood vessel walls are thickened, their lumens are narrowed, and even their blood vessels are occluded. This may be due to the increased production of growth factors such as vascular endothelial growth factor (VEGF) and fibroblast growth factor (FGF), which can stimulate the remodeling of blood vessel walls, resulting in a thickening of the basement membrane, which favors local deposition of proteins and lipids and promotes sclerosis and impaired vasodilation. Furthermore, chronic hyperglycemia acts on cerebral small blood vessels, leading to local blood flow regulation and metabolic disorders, in turn leading to decreased blood flow and ischemic injury of deep penetrating branch arteries. Atherosclerosis is the main cause of chronic cerebral ischemia. Severe atherosclerosis can lead to decreased blood flow in the distal blood supply and cause diffuse cerebral insufficiency and demyelination of the white matter. Besides, the result of vascular damage caused by this chronic disease is glial hyperplasia of the blood vessel walls around the ventricle, leading to demyelination of the white matter. In addition, diabetes is a well-known risk factor for cerebrovascular disease, as mentioned earlier, and it is related to glucose toxicity, abnormalities in cerebral insulin homeostasis, and microvascular abnormalities (4).

4. The oxidative stress in the body of diabetic patients is greater than that of non-diabetic patients, and the active oxidative products are increased accordingly. Consequently, the function of antioxidant system is weakened, and then microvascular diseases are more likely to occur. First, high glucose concentration increases oxidative stress by overproduction of the superoxide radical in the mitochondria (14), and this oxidative stress further impairs endothelial function (15, 16). However, oscillating glucose levels induce more oxidative stress than the high glucose concentration itself (17, 18). Aldose reductase catalyzes glucose to generate more fructose and sorbitol, which are deposited in the peripheral nerves in large quantities, further increasing the osmotic pressure in nerve cells and making them more prone to edema, degeneration, and necrosis. Oxidative stress caused by ROS overproduction plays a key role in the activation of other pathogenic pathways involved in diabetic complications, including elevated polyol pathway activity, non-enzymatic glycation, and PKC levels, which in turn lead to the development of microvascular complications (19–21). Hyperglycemia promotes the formation of ROS, which interacts with both deoxyribonucleic acid (DNA) and proteins, causing cellular damage, especially targeting mitochondrial DNA. The hyperglycemia and metabolic disturbance can increase the level of oxidative stress to further impair endothelia.

5. Increased systemic and cerebrovascular inflammation is one of the major pathophysiological features of type 2 diabetes and its cerebral vascular complications. The main mechanisms of hyperglycemia causing inflammatory response include NF-κB–dependent production of inflammatory cytokines, TLR expression, and increased oxidative stress (22). Some inflammatory proteins and cytokines increase the risk of WMHs by causing endothelial dysfunction. Among them, the activation of the classical complement system accelerates the hardening of arterioles, endothelial dysfunction, and other mechanisms in the cerebral microvessels, and finally causes pathological changes to cerebral microvessels and facilitates formation of WMHs.

6. A previous finding showed that the presence of WMHs-related type 2 diabetes is associated with elevated homocysteine concentration and insulin resistance (IR) (23). The mechanism by which hyperhomocysteinemia leads to WMH may be as follows: elevated levels of homocysteine in the blood induce oxidative damage to vascular endothelial cells and inhibit endothelial production of nitric oxide, which is a strong vasodilator. Moreover, hyperhomocysteinemia also promotes the growth of vascular smooth muscle cells, enhances platelet adhesion ability, and is associated with elevated levels of prothrombotic factors such as β-thromboglobulin and tissue plasminogen activator. In conclusion, higher homocysteine levels are associated with WMHs by damaging the endothelial function (24, 25).

It is known that IR is one of the pathogenic factors for type 2 diabetes. Although the specific mechanism of WMH with IR remains to be clarified, several mechanisms could be explained based on previous studies. First, IR is linked to WMHs by increased homocysteine levels. An animal study showed that insulin is involved in the regulation of plasma homocysteine concentrations by affecting the hepatic transsulfuration pathway, which is involved in the catabolism of homocysteine (26). Another animal study also indicated that IR is associated with elevated homocysteine concentrations and changes in two key enzymes in homocysteine metabolism, which subsequently lead to hyperhomocysteinemia (27). Second, IR can accelerate the development and progression of atherosclerosis by acting on its risk factors such as hypertension, hyperlipidemia, and obesity. Third, IR in association with lower cerebral perfusion in the frontal and temporal regions has been proposed (28). In addition, a study revealed that IR at the blood-brain barrier reduces the amount of glucose that can reach the brain, resulting in neuronal injury (29). In conclusion, it is possible that interactions among the incidence of WMHs, hyperhomocysteinemia, and IR are reinforced through mechanisms related to endothelial dysfunction.

7. Studies examining WMHs-related type 2 diabetes patients have a brain structural abnormalities basis. Because of the inconsistency between the WMHs and type 2 diabetes in previous studies, a study based on the automated segmentation method to offer precise, objective, and reproducible volumetric measurements of cerebral tissues in large numbers of patients, showed that type 2 diabetes was associated with a smaller volume of gray matter, larger lateral ventricle volume, and larger white matter lesion volume (30). These findings correspond to those of another study that reported that diabetic patients had greater WMH volume and brain tissue loss compared to non-diabetic patients (31). Some other studies have indicated that cognitive dysfunction was related to the reduced hippocampal volume (32, 33). In response to this finding, Milne et al. found that asymmetric hippocampal atrophy contributes to cognitive impairment in the incidence of WMHs with type 2 diabetes (34). De Bresser et al. compared the brain volumes of 55 diabetic patients with control participants over 4 years and found a greater increase in lateral ventricular volume over time in patients with type 2 diabetes (35). All of these reports suggest that the influence of diabetes on brain function is associated with WMHs, accompanied by damage to cognitive function. However, how diabetes causes changes in the brain structure is still speculative. Degenerative changes in cerebral small vessels may primarily contribute to this aspect, with other factors such as brain microvascular endothelial dysfunction, cerebrovascular inflammation, oxidative stress, and atherosclerosis caused by diabetes mellitus also playing a role.

8. Altered insulin signaling may be another contributory factor in WMH (36). An animal experiment revealed that reduced brain insulin signaling in mouse models of diabetes increased tau beta phosphorylation and amyloid beta peptide levels, both of which are associated with WMHs and cognitive decline (37).

9. A study indicated that glucose variability is associated with volume of WMHs (38). However, the hemoglobin A1c (HbA1c) level is not related to WMH volumes (35). It has been hypothesized that glycoalbumin/glycohemoglobin A1c (GA/HbA1c) is associated with WMH volumes in elderly diabetic patients The GA to HbA1c ratio (GA/HbA1c) is calculated by dividing GA by HbA1c, because GA/HbA1c can represent glucose variability with high sensitivity (39). The mechanism by which glucose fluctuation causes WMHs is another question that has interested researchers. Glucose variability is proven to induce oxidative stress and endothelial dysfunction, and acute glucose fluctuations are a greater trigger of oxidative stress than sustained hyperglycemia (40). As mentioned earlier, dysfunction of brain microvascular endothelial cells in type 2 diabetic patients promotes the occurrence of WMHs; hyperglycemia increases the level of oxidative stress to further impair the endothelia (41).

In summary, type 2 diabetes is an independent risk factor for WMHs. It can affect the white matter by affecting the regulation of cerebral blood flow and endothelial function, aggravating atherosclerosis, reducing the number of oligodendrocytes and other mechanisms, and finally leading to high signal intensity in the white matter. Type 2 diabetes causes WMHs through multiple pathways and mechanisms, which may interact with and promote each other. Therefore, active treatment of type 2 diabetes has certain clinical significance in the prevention of WMH. The interventions targeting the above mechanism are promising as they can reduce the risk of WMHs developing in patients with type 2 diabetes. However, the drivers of WMHs in patients with type 2 diabetes need further understanding and investigation.

## Conclusion

It is difficult to reverse WMHs in patients with type 2 diabetes; hence, blood glucose must be strictly controlled to prevent the occurrence and development of the disease. In future, targeted treatment for the pathogenesis of diabetes-related WMHs may bring new hope for preventing and delaying the occurrence and development of WMHs.

## Author Contributions

RL and GN designed the project. JS and BX wrote the paper. XZ revised the manuscript. ZH and ZL performed the literature search. All authors discussed and commented on the manuscript.

## Conflict of Interest

The authors declare that the research was conducted in the absence of any commercial or financial relationships that could be construed as a potential conflict of interest.
